# Relationship between Sprint Velocity and Peak Moment at Shoulder and Elbow in Elite Wheelchair Basketball Players

**DOI:** 10.3390/ijerph17196989

**Published:** 2020-09-24

**Authors:** Jorge Villacieros, Javier Pérez-Tejero, Guadalupe Garrido, Lena Grams, África López-Illescas, Amelia Ferro

**Affiliations:** 1 Department of Sports, Faculty of Physical Activity and Sport Sciences, Universidad Politécnica de Madrid, 28040 Madrid, Spain; jorge.villacieros@upm.es; 2 Department of Health and Human Performance, Faculty of Physical Activity and Sport Sciences, Universidad Politécnica de Madrid, 28040 Madrid, Spain; j.perez@upm.es (J.P.-T.); lupe.garrido.pastor@upm.es (G.G.); lena.grams@upm.es (L.G.); 3 Department of Health Sciences, Universidad Alfonso X “El Sabio”, 28016 Madrid, Spain; africa.lopez@aepsad.gob.es

**Keywords:** isokinetic, kinematic, speed, functional classification, performance

## Abstract

Specific wheelchair basketball (WB) skills on the court have been poorly analyzed in relation to improving players’ performance according to their functional class. The purpose of this study was to evaluate the associations between maximum velocity (Vmax) and peak moment (PM) in the shoulder and elbow joints in specific WB skills and to compare performance between the main two groups by functional class. Twelve male WB players, divided in categories A (functional classes 1.0–2.5) and B (class 3.0–4.5), performed a sprint test battery composed by four tests (with and without ball) and isokinetic tests. A significant relationship between PM of the internal and external shoulder rotation and the flexion and extension elbow with Vmax (*p* < 0.05) was found. During a 5 m backward sprint test, category B was faster in the last three meters than category A (*p* < 0.05) and also for the rest of the test but *p* = NS. Category B showed higher PM than category A for internal shoulder rotation (ISR) at 60 °/s and at 180 °/s on the dominant side (DS) (*p* < 0.05). In conclusion, ISR on the DS was found different for both groups and showed significant relationship with Vmax in all of the tests performed. Moreover, at higher speeds the elbow flexion and extension in DS were correlated with Vmax in all the actions with ball (passing, bouncing, and braking) reflected the importance of these joints movements in acquiring speed when performing sport specific WB skills.

## 1. Introduction

How to improve performance to train and to select players are the primary problems that concern coaches and researchers involved in wheelchair basketball (WB) competitions [1]. Worldwide, this is one of the most popular adapted sports for people with physical impairments. After eligibility process, players are allocated into five main functional classes: 1.0, 2.0, 3.0, 4.0, and 4.5 (a higher class denotes a higher volume of action and higher level of functional abilities on the court). In addition, players with some functional capabilities of the adjacent classes can be classified as 1.5, 2.5, or 3.5 for the International Wheelchair Basketball Federation (IWBF) [2], and players are grouped in category A (1.0 to 2.5, without hip control) or category B (3.0 to 4.5, with hip control). The sum of the points of the five players in one team on the court cannot exceed 14 points in total [2].

As a Paralympic modality, WB is under The International Paralympic Committee (IPC) Classification Code: A fundamental document upon with classification in Paralympic sports and it has to be applied by all members of the Paralympic Movement including athletes, international federations and international organizations of sport for people with disabilities [3], as, for instance, the IWBF. In this regard, all sport-specific eligibility (and classification) systems in Paralympic Sports, including WB, need to be evidence-based: It means that the system must have a clearly stated purpose and that empirical evidence must indicate that the methods used to identify eligible athletes will achieve the stated purpose [3,4]. In the case of WB classification system consensus, one proof of the acceptance of its classification system is that it has remained in use for more than 30 years with only small modifications [5], being the last 30 years the period of greatest expansion and professionalization of this sport. However, up to date, few studies has been developed to base the functional classification in WB with objective and evidence criteria [6]. Even more, the controversy between outcomes limits the future development, for instance, when dealing with the role of trunk stabilization in WB functional classification [7,8]. Also, most of the studies analyzing physiological and biomechanical features of this sport framed their results using the WB classification system [9], therefore it is accepted in the literature its use to provide practical application and guidance of the studies.

To gain insight into the specificity of this complex team sport, many studies have examined WB specific performance parameters developing different field test batteries dealing with several performance tests and abilities [10,11,12] but few of them focused in sprint ability using objective measurements [13,14]. For instance, in the study by De Groot et al. [10], a fitness test battery of 10 skill tests was specifically designed for WB fitness assessment. It was observed through a factor analysis that five of the ten WB skill tests focused mainly on speed, which would reflect its importance to perform in WB competition. In this line, it has been noted that sprint ability is a crucial task in WB [15]. Previous research focused on classification levels and athletes’ sprint capacity with different results [11,13]. For instance, de Lira et al. [16] registered correlations between functional classification of players and their level of anaerobic performance in terms of peak power, relative peak power and mean power. In another study, Ferro et al. [13], using a novel laser methodology for sprint assessment, found the higher speeds in the 2.0–2.5 players in a 20 m test. Previous findings show different results regarding the relationship between WB functional classification and sprint performance in WB players, so more research is needed in this topic.

For analysis purposes, wheelchair propulsion is normally divided into two main phases, propulsive phase and recovery phase. The propulsive phase is beginning with an internal shoulder rotation (ISR) and the recovery phase is initiated with an external rotation. If we observed at the elbow level, alternating flexion/extension is the typical pattern during propulsion [17]. The muscle strength of the arm flexors and extensors in paraplegic and non-paraplegic WB players was studied by Calmels et al. [18]. Their study indicated that muscle mass was increased in paraplegic subjects, but a correlation between muscle mass and strength was only found in non-paraplegic subjects [18]. In this regard, Basar et al. [19], who studied the shoulder rotator strength of young national and junior national male WB players using isokinetic tests, found differences in peak moment (PM) between the young and junior national players. In another recent study, relationships between strength-power values and velocity (using a 20 m sprint test) were found in a sample of non-elite WB players [20], suggesting that differences in explosiveness and absolute strength could account for differences between players at different levels of national competitions. These results could be very useful for coaches and technical staff to structure strength and conditioning training to improve sprint capacity, as it has been selected as one of the most important components of WB competition [13].

Our study focused in the assessment of WB elite players’ sprint ability and their performance in selected sport specific skills which are key to WB performance, such as starting, sprinting, and braking [21] with and without the ball. To the best of our knowledge, sprint capacity assessment in WB and its relationship with shoulder and elbow strength during wheelchair propulsion remains unknown in elite WB population. For this reason, the aims of this study were (a) to evaluate the associations between a newly designed WB field sprint tests battery and isokinetic tests at shoulder (internal and external rotation) and elbow (flexion and extension) joints in a sample of elite WB players; and (b) to assess possible differences according to functional classification for both aforementioned evaluations.

## 2. Materials and Methods

### 2.1. Participants

Twelve male WB players participated in the study. All of them were elite WB players (recognized by the Spanish High Council of Sport and Paralympic Committee) from the Spanish National WB squad at the time of the study. They were free of injury and trained 4–5 days per week. The study was undertaken during the competitive period prior to main annual competition. Descriptive participant´s information is presented in Table 1. The University Ethics Committee approved the study (UPM-04-02-2015) and it was undertaken according to the Helsinki Declaration on research in humans [22].

### 2.2. Instrumentation

A laser sensor-type 1 Jenoptik LDM301 (Jena, Germany) was used to measure distances with a range of 0.5–300 m, accuracy of ± 0.06 m for 2 kHz and a resolution of 0.001 m. The data were recorded and processed with DasyLab software, v. 10.0 (Data Acquisition System Laboratory of National Instruments, Mönchengladbach, Germany) to obtain maximum velocity (Vmax) and average velocities (Va) at 200 Hz. These components were integrated into a Kinematic analysis system in real time for training and sports competitions (UPM-UPO, Madrid, Spain) [23], BioLaserSport^®^ [24] whose validity and reliability were calculated previously [25]. Shoulder and elbow muscle strength was evaluated using an isokinetic Biodex^®^ dynamometer *Multi-Joint System-PRO* (Biodex^®^ Corp., Shirley, NY, USA) obtaining PM and total work (TW).

### 2.3. Design and Procedures

A test battery was designed considering the opinion of coaches from the Spanish National Men Team squad. Several pilots per tests were performed with coaches and players in order to define the final protocol, adequacy and their real utility. Following the coaches and players criteria and the ecological validity, the sport specific skills domains selected were starting, sprinting, braking, and ball handling. Field sprint test were carried out on one day between 9:00 and 13:00 h and the isokinetic tests were carried out the next day at the same time.

#### 2.3.1. Field Sprint Tests

**Figure 1:**
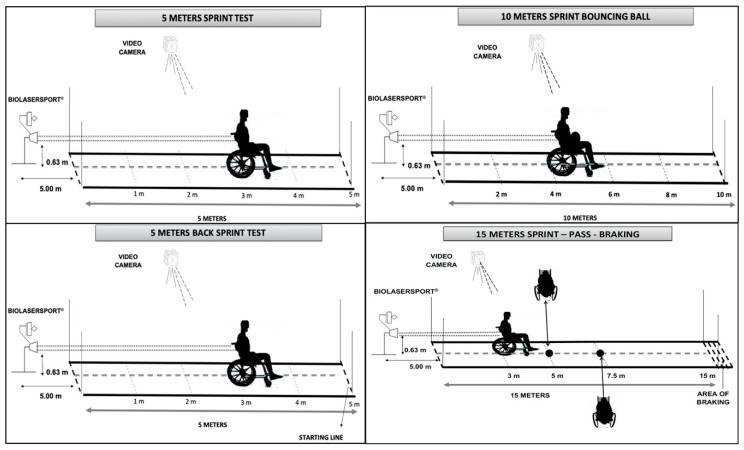
Diagrams of the battery of field sprint tests.

5 m forward sprint test (5F). The participant started from a stationary position, with the front wheels behind the starting line, pushing 5 m forward as quickly as possible. The court was divided in five sections (0–1 m, 1–2 m, 2–3 m, 3–4 m, and 4–5 m). The test domain was (start-up) speed. The reliability of the test was assessed with the intraclass correlation coefficient (ICC), showing high values (0.88 for Vmax and 0.91 for Va) (Figure 1).

10 m sprint forward bouncing ball test (10FB). The participant started with a ball from a stationary position and pushes 10 m as rapidly as possible, adhering to the IWBF rules for dribbling. The test was divided into five sections (0–2 m, 2–4 m, 4–6 m, 6–8 m and 8–10 m). The test domains were speed and ball handling while bouncing. The ICC was 0.81 for Vmax and 0.87 for Va (Figure 1).

5 m backward sprint test (5B). The participant started from a stationary position and pushes 5 m backward as quickly as possible. The test was divided in five sections (5–4 m, 4–3 m, 3–2 m, 2–1 m, and 1–0 m). The test domain was (start-up) backward speed. The ICC was 0.80 for Vmax and 0.88 for Va (Figure 1).

15 m sprint-pass-braking test (15PB). The participant started from a stationary position in possession of the ball, with the front wheels behind the start line, and he pushed 15 m as quickly as possible. At 5 m from the starting line, the participant had to brake and pass the ball, speeding up until 7.5 m, braking again and receiving from a partner the ball, and must attain maximum speed until 15 m and then, braking again. The test was divided in five sections (0–3 m, 3–5 m, 5–7.5 m, 7.5–15 m, and 15-braking distance m). The test domains were speed, pass, ball handling and braking. The ICC was 0.86 for Vmax and 0.90 for Va (Figure 1).

The tests application and data collection were performed according to Ferro et al. [13]. It was made on a wooden surface and the tubes of the wheelchairs were inflated up to the maximum. The test started with a standard 15 min warm-up. The starting signal was verbal and used the words “ready” and then “when you like”, so undesirable effects of players’ reaction times on sprint performance were avoided. All of the tests were performed two times, and the end score was the mean of the two trials (Figure 1).

#### 2.3.2. Isokinetic Testing

Internal shoulder (ISR) and external shoulder rotation (ESR). The participants were positioned in a seated position with the trunk stabilized uniformly and with 45° shoulder abduction in the scapular plane. The elbow was supported in 90° flexion, and the forearm and wrist were in neutral pronation-supination. Auto-adhesive straps were placed horizontally across the chest and pelvis to stabilize the trunk to the seat. The participants were tested through a 70° range of motion.

Elbow flexion (EF) and elbow extension (EE). The participant was placed in the seated position with the shoulder in abduction at 45°. The elbow was supported by a lateral cushion, which did not limit arm extension. The participants were tested through an 80° range of motion and the arm and the testing apparatus were statically weighed to provide gravity compensation data, and corrections were incorporated [26].

The two shoulders and two elbows, on the dominant side (DS) and non-dominant side (NDS), were tested in random order. A 10 min global warm-up consisted in three submaximal repetitions at each angular velocity of the test in the isokinetic machine was performed. Data were obtained successively in the concentric contraction mode at 60 °/s and 180 °/s for the shoulders and 60 °/s and 150 °/s for the elbows with 5 and 10 repetitions, respectively. Each series was separated by 60 s rest. The values of PM and TW were registered. The participants were given verbal encouragement but no visual feedback from the PC screen was provided.

### 2.4. Statistical Analysis

Descriptive analyses of each test were performed and normal distribution was tested with the Shapiro–Wilk test. The relationship between PM and Vmax, assessed with the different tests, was examined using Pearson’s product–moment correlation coefficient (r). Student’s paired t-test was applied to assess possible differences between the DS and NDS and between ESR and ISR and between FE and EE. An independent t-test was used to examine the difference in variables between A and B groups. The confidence interval (CI) at 95% and Cohen’s *d* was determined to estimate the effect size. Effect sizes (d) greater than 0.8, between 0.8 and 0.5, between 0.5 and 0.2 and less than 0.2 were considered as large, moderate, small and trivial, respectively [27]. In order to correctly detect the difference between average of the two samples, a post hoc power analysis (P) also was calculated with G*Power software, v. 3.1.9 (Psicho.uni.Düsseldor.de, Düsseldor, Germany). The significance level was determined at *p* < 0.05. The rest of the calculations were performed with the SPSS software program, v. 21.0 (IBM Corp., Armonk, NY, USA).

## 3. Results

### 3.1. Field Sprint Tests

**Figure 2:**
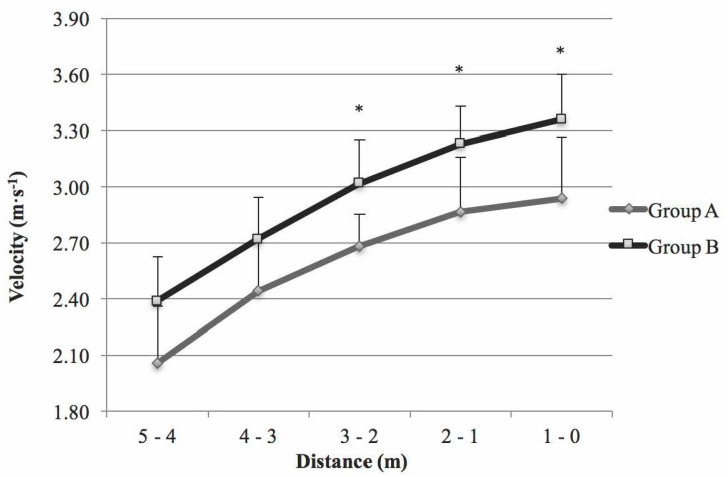
Maximum velocity (Vmax) by sections between functional classification in the 5 m sprint back test (* *p* < 0.05).

Descriptive data for every test concerning A and B groups are presented in Table 2. Group B was faster in the 5B for Vmax3–2 m (*p* < 0.05, d = 0.65, CI = 2.70–3.04, *p* = 0.80), Vmax2–1 m (*p* < 0.05, d = 0.63, CI = 2.88–3.26, *p* = 0.80) and Vmax1–0 m (*p* < 0.05, d = 0.63, CI = 2.96–3.40, *p* = 0.83) than group A (Figure 2). In the rest of the sprint tests, differences were not statistically significant between groups; however, for Vmax, group B showed higher results than group A. In the 5F, group B was 4.77% faster than group A; in 15PB test, group B was 7.78% faster and in the 10FB, group B was 3.13% faster.

### 3.2. Isokinetic Test

The results showed significant differences between group B and group A for ISR in PM at 60 °/s in DS (*p* < 0.05, d = 0.71, CI = 61.14–88.86, p = 0.82) and at 180 °/s in DS (*p* < 0.05, d = 0.67, CI = 53.85–83.65, *p* = 0.81) (Table 2). Regarding TW for ISR, there were significant differences at 60 °/s in DS (*p* < 0.05, d = 0.69, CI = 281.28–429.05, *p* = 0.85) and at 180 °/s in DS (*p* < 0.05, d = 0.65, CI = 422.77–710.89, *p* = 0.75) and NDS (*p* < 0.05, d = 0.62, CI = 451.56–681.61, *p* = 0.70). Neither for elbow joint nor for ESR there were significant differences (*p* = N.S.). Non-significant differences were found in DS and NDS.

### 3.3. Relationships between Peak Moment and Sprint Velocity

ISR in DS at 60 °/s and 180 °/s showed correlations with Vmax in 5F test in all sections (except at 180 °/s in Vmax1–2); in 10FB test in all sections except in 0–2 m and in 15PB test in all sections except in 3–5 m (Table 3). In 5B test for ISR in DS at 60 °/s showed correlations with Vmax in all sections except 5–4 m and 4–3 m and at 180 °/s only had correlations with Vmax3–2. In 10 FB test ERS in NDS showed correlations with Vmax in all sections, except in the 0–2 m and in DS except in 0–4 m. In sections 8–10 in 10 FB and in 5–7.5 and 7.5–15 m in 15 PB, EF, and EE in DS showed correlations with Vmax while EE in NDS only in the both last sections (Table 3).

## 4. Discussion

According to our knowledge, the aim of this study is original, as it is the first to correlate PM at the joint level with sprint capacity, a key sport-specific skill, in a sample of elite WB players, assessing whether there was any difference between their functional classification. The main contributions of the present study were the significant associations discovered between Vmax achieved by WB players with specific designed field sprint tests and PM obtained in specific on court movements (ISR, ESR, EE, and EF) using an isokinetic dynamometer (Table 3). Significant differences were found regarding Vmax between group A and B only in the 5B, with better results obtained in group B in the last 3 m (Figure 2). Also, the relationships between ISR and with Vmax in the last 3 m on 5B were found (Table 3). To support these results, it was calculated power analysis obtaining a value above 0.8, being the minimum value recommended in biomedical studies [27]. Although, for the rest of tests, no significant differences were found between the two groups, players with the ability to stabilize their trunk actively (category B) presenting better results (Table 2). Several authors have related functional classification with different parameters, for instance, Vanlandewijck et al. [28] observed that the upper classifications (category B, 3–4.5 classes) had better results in total amount of actions performed per time played, while Molik et al. [29] showed that athletes with active pelvic control (category B) demonstrated greater peak power output and mean power output than the group without control (category A, 1.0–2.5 classes) in an anaerobic Wingate test. Our findings were in line with previous literature, in which upper WB classes (3 to 4.5) had better results, but no significant differences were found between the two groups for field tests sprint performance [9,13,14]. Even more, to our knowledge no studies have been performed to accurately describe the velocity curve of WB for the 5F, neither for the tests presented in this research. The relation with the capability of PM generated by the shoulder and elbow is also unknown. In this line, technological advancement can offer new alternatives to improve knowledge applied to functional classification in wheelchair sports´ specific skills [6,30], as WB is.

In most wheelchair sports, general and specific strength training is a critical component for success in competition [31]. Thus, it is logical to believe that WB depends on strength and power in the upper extremities [20]. However, regarding functional classification, previous studies did not observe differences in PM among classes (classes 1, 2, and 3) or in external and internal rotation [32]. These results differed from those obtained in our study, in which significant differences between group B and group A were observed for ISR relative to PM (Table 2). Other studies compared, based on isokinetic test differences, young national players and national junior players and groups of different ages, older and younger national players, on which the PM and TW values were significantly greater in group 1 (young national player) for ISR in DS at 60 °/s [19]. In another study, Granados et al. [20] found that the absolute strength and explosiveness of the upper extremity muscles were higher in the first division team than in the third division team in handgrip, maximal pass and medicine ball throw, indicating that high absolute values of strength and muscle explosiveness could be required for successful performance in high level WB. In this regard, relationships between PM in shoulder and elbow and Vmax achieved by players were observed in our study. In the 5F test, in which the ability to sprint without any technical action was assessed, it was chiefly noted that the ISR of the DS presented a significant relationship in each section with Vmax when working at 60 °/s (values above r = 0.672) and at 180 °/s (except in 1–2 m) (Table 3). This relationship of force and velocity in the wheelchair seems logical, considering that ISR starts at the beginning of propulsion phase [17].

The Vmax of 10FB test in each section of the test, except for the first section, showed significant correlations with ISR in DS for both velocities (Table 3). This could be explained because, at the start of the test, players must throw the ball to free their hands and propel the wheelchair. This situation did not occur without the ball in the 5F test. ESR in DS and NDS at 180°·s^–1^ also showed relationships with Vmax in all sections of the sprint test except the first two meters, which could explain the differences when using the ball (bouncing with one hand while the other hand pushes the wheelchair, for example) involving a greater number of shoulder and upper trunk movements. In our opinion, because of this involvement, EF and EE were correlated at velocity of 150 °/s with Vmax (except EF in NDS) in the last section when they achieved their Vmax, due to the necessity to move the ball and the wheelchair simultaneously (Table 3).

For the 15PB test, during the section in which the players braked (3–5 m), the relationship between ISR in DS with Vmax for both, 60 °/s and 180 °/s, was lost (Table 3). Similar to the previous test, this movement in DS was again influenced probably by speed gain. ISR in NDS and ESR on both sides also showed significant correlations with Vmax at 180 °/s, which could be explained by the braking actions being performed by muscles that involve shoulder rotation. The alternating elbow movement of flexion and extension at 150 °/s influenced by the use of implements (passing the ball) and actions (such as braking) the wheelchair is special significant in the last sections where higher Vmax were performed. So, the NDS of the ESR and EE only were significant in actions with ball (Table 3).

Following Vanlandewijck et al. [21], the ability to accelerate the wheelchair from standstill is determined by several components, classified in three major groups: Those related to the wheelchair configuration (such as the overall rolling resistance and internal friction); those related to the player (such as explosive strength and propulsion technique); and those related to the adjustment of the wheelchair characteristics to the functional abilities of the player (wheelchair-user interface). This study focused in those related to the player, so results should be interpreted in that line. Also, in this study, as previously indicated, one important limitation was the small sample size. A larger sample would probably have allowed for better profiling of every WB class. For this reason, statistical calculations were made to give more reliability to our results, like CI and power analyses. However, all of the players belonged to the Spanish National WB squad and were recognized by the Spanish High Council of Sport and Paralympic Committee, reporting a high value to the results. Another limitation of the study was not to taking into consideration players´ trunk involvement, as it has been demonstrated it is related with functional classification [8]. In this line, we found also significant differences at ISR level, but for the field tests, only for the 5B test these differences were found (Table 2). So in our opinion, one possible explanation of these significant differences found in this test could be explained by trunk involvement per functional class.

Future studies should focus on force training of the upper limbs and movement control and speed during WB sport specific skills, examining the influence of other specific movements (human trunk) on WB player sprint capacity. Furthermore, these studies should focus on the development of specific training programs considering the importance of the ISR of the DS to sprint capacity, to avoid injuries and muscle imbalances. Although the importance lies in the DS, it should be noted that the work considered both sides, as well as in the work of agonists and antagonists, to prevent muscle imbalances. Studies on Female Spanish National WB for analyzing gender differences would be interesting.

## 5. Conclusions

In conclusion, WB players from category B (3.0–4.5 classes) were significantly faster than group A (1.0–2.5 classes) players for 5B test. For the rest of the tests, WB players from category B were faster in 15PB, 10FB, and 5F tests in comparison to their category A counterparts, although those differences were not significant. Also, the role of ISR on the DS and its significant relationship with Vmax in all of the tests performed reflected the importance of this movement in acquiring faster sprint capacity when using the wheelchair with and without the ball and in a straight line. At higher velocities, the elbow flexion and extension in dominant side were relevant in actions with ball as bouncing, passing and braking. Moreover, the non-dominant side of the external shoulder rotation and extension elbow were only significant during these actions. These findings emphasize the need to carry out analyses focused on improving sport performance considering the functional classification in the WB, as a Paralympic sport, in which sprint ability and applied strength are relevant skills that must be specifically coached.

## Figures and Tables

**Table 1:** Wheelchair basketball players’ characteristics.

Group	Age	Weight (Kg)	Height (m)	Disability	IWFB Classification	Experience (Years)
A	23	48	1.75	Paraplegia	1	6
A	41	69	1.76	Paraplegia	1	19
A	39	78	1.84	Paraplegia	1.5	22
A	22	59	1.84	Paraplegia	2.5	5
A	23	72	1.75	Paraplegia	2.5	6
B	26	73	1.70	Spina Bifida	3	8
B	26	83	1.78	Spina Bifida	3	8
B	39	90	1.82	Paraplegia	3	21
B	28	52	1.71	Spina Bifida	3	10
B	23	75	1.86	Amputation	4	5
B	32	102	1.80	Amputation	4	17
B	37	87	1.88	Amputation	4	20
Sample	29.91 ± 7.27	75.66 ± 13.97	1.79 ± 0.05			12.25 ± 6.90

IWFB = International Wheelchair Basketball Federation.

**Table 2:** Descriptive data of the tests performed by wheelchair basketball players.

Field Test (Vmax) (m/s)	Category	Mean	SD	SE	*p*	d
5 m forward sprint (5F)	A	3.79	0.20	0.09	0.08	0.4
	B	3.98	0.14	0.05		
10 m forward sprint with ball (10FB)	A	4.03	0.31	0.14	0.461	0.21
	B	4.16	0.29	0.11		
5 m back sprint (5B)	A	2.81	0.34	0.15	0.028 *	0.63
	B	3.06	0.30	0.11		
15 m sprint-pass-braking (15PB)	A	4.03	0.34	0.15	0.072	0.49
	B	4.37	0.25	0.10		
Isokinetic testing PM (Nm) Dominant side (DS)						
Internal shoulder rotation (ISR) 60°/s	A	57.40	15.57	6.96	0.09 *	0.71
	B	87.57	16.39	6.19		
180°/s	A	51.00	21.67	9.69	0.018 *	0.67
	B	81.43	15.67	5.92		
External shoulder rotation (ESR) 180°/s	A	39.40	12.93	5.78	0.151	0.42
	B	52.57	15.40	5.82		
Elbow flexion (EF) 60°/s	A	73.20	15.30	6.84	0.52	0.19
	B	80.14	19.18	7.25		
150°/s	A	62.00	16.23	7.26	0.703	0.11
	B	66.00	18.12	6.85		
Elbow extension (EE) 60°/s	A	73.80	17.44	7.80	0.565	0.17
	B	79.86	17.34	6.56		
150°/s	A	58.40	17.49	7.82	0.293	0.3
	B	69.86	17.73	6.70		

Vmax: Maximum velocity; PM: Peak moment; Category A—class 1.0–2.5 (7 athletes); category B—class 3.0–4.5 (5 athletes); * statistically significant difference (*p* < 0.05); large effect size (Cohen’s d > 0.50); SD—standard deviation; SE—standard error.

**Table 3:** Correlations between maximum velocity in the field sprint tests and peak moment joint in isokinetic test (note: It shows only the variables groups with some correlations).

Field Test	Vmax by Sections (m/s)	EF 60 °/s DS	EF 150 °/s DS	EE 150 °/s DS	EE 150 °/s NDS	ISR 60 °/s DS	ISR 180 °/s DS	ISR 180 °/s NDS	ESR 180 °/s DS	ESR 180 °/s NDS
	Vmax0–1					0.672 *	0.599 *			0.587 *
	Vmax1–2					0.676 *	0.557			0.484
5F	Vmax2–3					0.775 **	0.689 *			0.581 *
	Vmax3–4					0.719 **	0.628 *			0.562
	Vmax4–5					0.677 *	0.579 *			0.556
	Vmax5–4					0.411	0.481			
	Vmax4–3					0.485	0.443			
5B	Vmax3–2					0.646 *	0.610 *			
	Vmax2–1					0.579 *	0.509			
	Vmax1–0					0.621 *	0.490			
	Vmax0–2		0.408	0.280	0.256	0.574	0.510		0.375	0.364
	Vmax2–4		0.523	0.528	0.470	0.675 *	0.648 *		0.556	0.589 *
10FB	Vmax4–6		0.556	0.559	0.500	0.745 **	0.677 *		0.606 *	0.641 *
	Vmax6–8		0.545	0.556	0.560	0.749 **	0.731 **		0.640 *	0.650 *
	Vmax8–10		0.644 *	0.683 *	0.634 *	0.673 *	0.643 *		0.619 *	0.635 *
	Vmax0–3	0.594 *	0.505	0.475	0.553	0.707 *	0.638 *	0.583 *	0.584 *	0.654 *
15PB	Vmax3–5	0.462	0.405	0.416	0.507	0.478	0.443	0.575	0.495	0.635 *
	Vmax5–7.5	0.580 *	0.582 *	0.582 *	0.536	0.650 *	0.601 *	0.522	0.547	0.574
	Vmax7.5–15	0.742 **	0.660 *	0.675 *	0.719 **	0.744 **	0.777 **	0.659 *	0.656 *	0.668 *

5F: 5 m forward sprint test; 5B: 5 m backward sprint test; 10FB:10 m forward sprint bouncing ball test; 15PB: 15 m sprint-pass-braking tests; IRS: Internal shoulder rotation; ERS: external shoulder rotation; EF: Elbow flexion; EE: Elbow extension; DS: Dominant side; NDS: Non-dominant side. (* *p* < 0.05, ** *p* < 0.01).
